# Leaf onset in the northern hemisphere triggered by daytime temperature

**DOI:** 10.1038/ncomms7911

**Published:** 2015-04-23

**Authors:** Shilong Piao, Jianguang Tan, Anping Chen, Yongshuo H. Fu, Philippe Ciais, Qiang Liu, Ivan A. Janssens, Sara Vicca, Zhenzhong Zeng, Su-Jong Jeong, Yue Li, Ranga B. Myneni, Shushi Peng, Miaogen Shen, Josep Peñuelas

**Affiliations:** 1 Key Laboratory of Alpine Ecology and Biodiversity, Institute of Tibetan Plateau Research, Center for Excellence in Tibetan Earth Science, Chinese Academy of Sciences, Beijing 100085, China; 2 CAS Center for Excellence in Tibetan Plateau Earth Sciences, Chinese Academy of Sciences, Beijing 100085, China; 3 Sino-French Institute for Earth System Science, College of Urban and Environmental Sciences, Peking University, Beijing 100871, China; 4 Department of Ecology and Evolutionary Biology, Princeton University, Princeton, New Jersey 08544-1003, USA; 5 Department of Biology, University of Antwerp, Universiteitsplein 1, Wilrijk 2610, Belgium; 6 LSCE, UMR CEA-CNRS, Bat. 709, CE, L'Orme des Merisiers, Gif-sur-Yvette F-91191, France; 7 Jet Propulsion Laboratory, California Institute of Technology, Pasadena, California 91011, USA; 8 Department of Earth and Environment, Boston University, 675 Commonwealth Avenue, Boston, Massachusetts 02215, USA; 9 CREAF, Cerdanyola del Valles, Barcelona 08193, Spain; 10 CSIC, Global Ecology Unit CREAF-CSIC-UAB, Cerdanyola del Valles, Barcelona 08193, Spain

## Abstract

Recent warming significantly advanced leaf onset in the northern hemisphere. This signal cannot be accurately reproduced by current models parameterized by daily mean temperature (*T*_mean_). Here using *in situ* observations of leaf unfolding dates (LUDs) in Europe and the United States, we show that the interannual anomalies of LUD during 1982–2011 are triggered by daytime (*T*_max_) more than by nighttime temperature (*T*_min_). Furthermore, an increase of 1 °C in *T*_max_ would advance LUD by 4.7 days in Europe and 4.3 days in the United States, more than the conventional temperature sensitivity estimated from *T*_mean_. The triggering role of *T*_max_, rather than the *T*_min_ or *T*_mean_ variable, is also supported by analysis of the large-scale patterns of satellite-derived vegetation green-up in spring in the northern hemisphere (>30°N). Our results suggest a new conceptual framework of leaf onset using daytime temperature to improve the performance of phenology modules in current Earth system models.

## Abstract (web summary)

Recent warming has significantly advanced leaf onset in the northern hemisphere. Here, the authors show asymmetric effects of daytime and nighttime temperature change on the timing of leaf onset.

## Graphical abstract

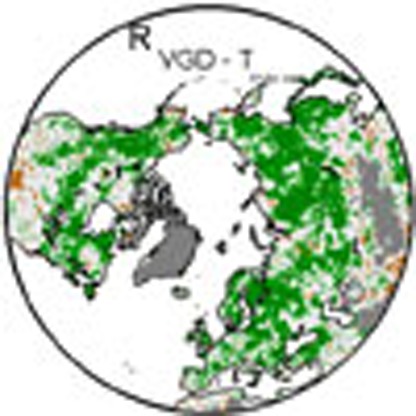

Phenology, the timing of periodic events in the life cycle of living organisms, is sensitive to climate^1^,^2^,^3^,^4^. Phenological changes induced by climate change can alter species interactions^5^,^6^ and ecosystem functioning, resulting in changes in the carbon, water and energy balances and, hence, climatic feedbacks^7^. Data from satellite greenness indices, field observations, and atmospheric CO_2_ observations all show a trend towards an earlier spring green-up for northern vegetation over recent decades, super-imposed on high interannual variability^1^,^2^,^8^,^9^. Spring temperature correlates well with this trend and with the interannual variability of spring green-up^2^,^8^. Mean temperature is the principal variable used by dynamic global vegetation models (DGVMs) for calculating leaf onset in temperature-limited biomes. These models, however, simulate onset dates that have large systematic errors compared with the *in situ* and satellite observations^3^,^10^, suggesting limitations in their equations describing phenology.

In cold and temperate regions, plants generally require the accumulation of a certain amount of heat to trigger spring leaf onset. Several studies also outline the need for plants to endure cold conditions during their dormancy, which defines chilling requirements^11^,^12^. Yet, evidence for a widespread chilling requirement is thin, and statistical models without chilling can predict the leaf onset date. Growing degree days (GDDs), the sum of daily mean temperature (*T*_mean_) above a fixed threshold value, is a common surrogate for the accumulation of heat needed to unfold leaves^13^. Current phenological models that use daily mean temperature ignore potentially different responses of plants to daytime and nighttime warming (see,for example, refs 14, 15). In other words, if daytime and nighttime temperatures impact distinctly the heat requirement of GDD, statistical and conceptual models of leaf onset must carefully distinguish which temperature should be used. In addition, global warming is increasing nighttime temperatures more than daytime temperatures, which makes the use of mean daily temperature likely impractical for modelling phenology^16^.

Here, using vegetation green-up date (VGD) diagnosed from satellite observations and *in situ* observations of leaf unfolding dates (LUDs) in Europe and the United States, we demonstrate that the interannual anomalies of the timing of leaf onset are triggered by daytime more than nighttime temperature across the northern hemisphere.

## Results

### Evidence from *in situ* observation

We first compared daytime versus nighttime temperature accumulation for predicting *in situ* observations of LUD in Europe and the United States over the past 30 years (1982–2011). Twenty-four plant species from 2,400 phenology sites in Europe and lilac (*Syringa* L.) shrubs from 35 phenology sites in the United States were selected from the European Pan European Phenological Database (hereafter EU) and the USA National Phenology Network (hereafter US) (Supplementary Fig. 1; see Methods), respectively. Temperature data included monthly averaged daily maximum (*T*_max_) and minimum temperature (*T*_min_) with a spatial resolution of 0.5° obtained from the Tyndall Centre Climate Research Unit (CRU TS 3.20; see Methods). Both precipitation and cloudiness were included in the partial-correlation analyses, while other variables such as soil moisture and soil temperature were not included, since temperature co-varies with these variables.

**Figure 1:**
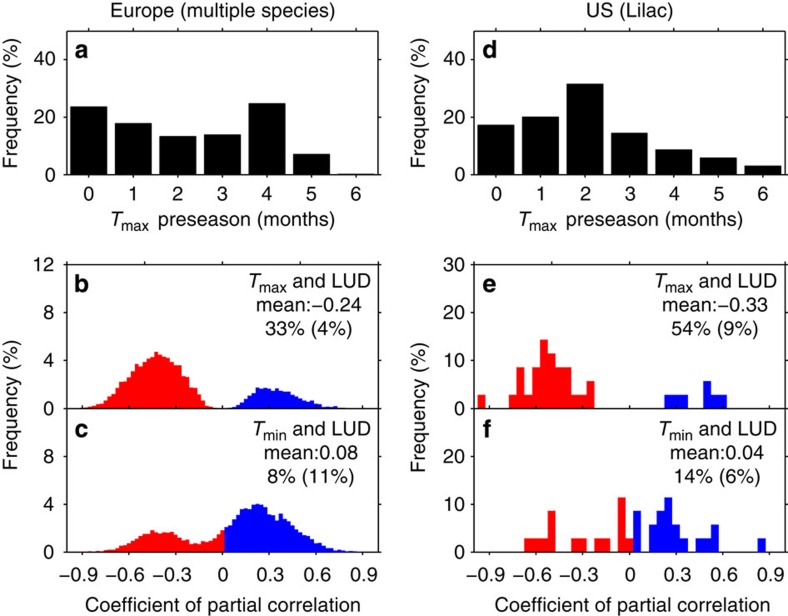
Responses of *in situ*-observed LUDs to *T*_max_ and *T*_min_ in Europe and the United States during 1982–2011. The frequency distributions of the length (in months) of *T*_max_ preseason in (**a**) Europe and (**d**) the United States are shown. The *T*_max_ preseason is defined as the period with the highest negative partial correlation between LUD and averaged *T*_max_ for the months preceding LUD. Frequency distributions of the highest partial-correlation coefficients between LUDs and preseason *T*_max_ in (**b**) Europe and (**e**) the United States after controlling for corresponding *T*_min_, cloudiness and precipitation. Frequency distributions of partial-correlation coefficients between LUD and *T*_min_ in (**c**) Europe and (**f**) the United States during the same preseason as in a after controlling for corresponding *T*_max_, cloudiness and precipitation. Note that LUDs of multiple species in Europe and only lilacs (*Syringa* L.) in the United States were analysed. The mean values of partial-correlation coefficients across all phenological stations, the percentages of significantly negative partial correlations and the percentages of significantly positive partial correlations (in parentheses) are provided in **b**,**c**,**e** and **f**.

Many lines of evidence show that spring LUD is strongly correlated with temperature of the preceding months (preseason)^8^. Here we define the length of the preseason for *T*_max_ by *L*_max_ (and by *L*_min_ for *T*_min_). The value of *L*_max_ is calculated for each site as the period before LUD for which the partial-correlation coefficient between LUD and *T*_max_ is maximized in absolute value (controlling for the effects of T_min_, precipitation and cloudiness; note that the correlation is negative; see Methods) (Supplementary Fig. 2). *L*_max_ ranges from 0 to 3 months across most of the species–site–year combinations for both the phenology data sets (68% for EU and 83% for US; Fig. 1a,d). For the EU network, the partial interannual correlation between LUD and *T*_max_ averaged during *L*_max_ is negative and significant (*P*<0.05) at 33% of the species–site combinations. By contrast the significantly negative partial interannual correlation between LUD and *L*_max_-averaged *T*_min_ occurs at <8% of the species–site combinations, as limited as the significantly positive counterpart (Fig. 1b,c). Similarly, the partial interannual correlations between LUD and *L*_max_-averaged *T*_max_ were found significantly negative at 54% of the US lilac sites (*n*=35) compared with only 14% if *L*_max_-averaged *T*_min_ is used (Fig. 1e,f). Similar results were also found with *L*_min_-averaged variables (Supplementary Fig. 3; see Methods). This observation suggests a predominant role of *T*_max_ rather than *T*_min_ in controlling the interannual variations of LUD in both EU and the US phenological *in situ* data.

To further test the robustness of the results shown in Fig. 1, we performed the same analyses with climate data of weekly and biweekly resolution (see Methods). All analyses produced similar results as shown in Fig. 1 (Supplementary Figs 4–6), confirming the stronger relationship of LUD with daytime temperature rather than with nighttime temperature, which was not affected by the temporal resolution of the climate data sets. Furthermore, we also extended the analyses to include winter temperature as a predictor to account for chilling effects (see Methods). Here winter temperature was defined as the average *T*_mean_ during the period from the onset of the preceding dormancy (the time at which daily mean temperature falls below 0 °C, or the default date of 1 November in the year preceding LUD) to the beginning of the *T*_max_ preseason. Including winter temperature did not alter the conclusion that *T*_max_ is a stronger predictor of LUD than *T*_min_ (Supplementary Fig. 7).

### Evidence from satellite observation

**Figure 2:**
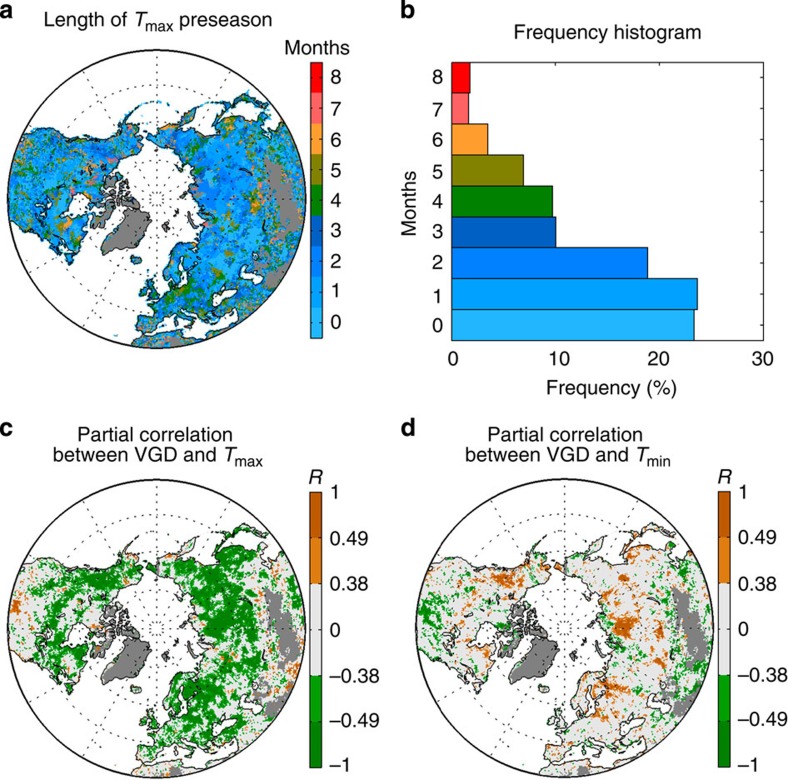
The relationship of the satellite-derived onset dates of vegetation green-up with *T*_max_ and *T*_min_ in the northern hemisphere during 1982–2011. (**a**) The spatial pattern of the length (in months) of the preseason defined as the period with the highest negative partial correlation between VGD and averaged *T*_max_ for the months preceding VGD. (**b**) The frequency distribution of the length of the preseason shown in **a**. (**c**) Partial-correlation coefficients (*R*) between preseason *T*_max_ and VGD after controlling for corresponding *T*_min_, cloudiness and precipitation. (**d**) Partial-correlation coefficients (*R*) between *T*_min_ and VGD during the *T*_max_-derived preseason after controlling for corresponding *T*_max_, cloudiness and precipitation. The 1% and 5% significance levels of the partial correlations correspond to ±0.49 and ±0.38, respectively.

*In situ* phenology observations cover only a small fraction of world's vegetation types, geographic ranges and climate gradients. To evaluate the generality of the *in situ*-observed asymmetric temperature effects on leaf onset in Europe and parts of the US, we further analysed the effects of daytime and nighttime temperature changes on satellite-derived VGD in the terrestrial northern hemisphere (> 30°N) over the past 30 years (1982–2011; see Methods). VGD at 0.5 × 0.5° resolution was estimated from time series of the NDVI3g data set (1982–2011) developed by the Global Inventory Modeling and Mapping Studies (GIMMS) group (see Methods). Note the reported VGD here is the average value from four different VGD algorithms: Spline Midpoint, Hants Maximum, Polyfit Maximum and Timesat SG (Savitzky–Golay; see Methods). Similar to the *in situ*-observation results, satellite-derived *L*_max_ ranged between 0 and 3 months across 76% of the study area (Fig. 2a,b), also in agreement with earlier findings^17^. Statistically significant (*P*<0.05) negative partial correlations between VGD and *L*_max_-averaged *T*_max_ were found in 42% of the study area (Fig. 2c). In contrast, over the same preseason only 11% of the study area showed significantly negative partial correlations between VGD and preseason averaged *T*_min_, mostly in temperate dry regions (Fig. 2d). Even when using the *T*_min_ preseason, the negative partial correlation between *T*_min_ and VGD remained less prevailing (13% of the study area exhibited significantly negative correlation coefficients, Supplementary Fig. 8b) than that between *T*_max_ and VGD (32% of the study area; Supplementary Fig. 8a).

In addition, we tested the robustness of the satellite-derived results using the four different satellite-derived VGD algorithms instead of the mean VGD from all algorithms (Supplementary Fig. 9), using different climatic data sets that had different time resolutions (Supplementary Figs 10–12) and taking chilling effects into account (Supplementary Fig. 13). All the tests returned similar results. In particular, we analysed data from individual meteorological stations (Supplementary Fig. 14) thereby avoiding any potential bias of spatial extrapolation like in the gridded climatic data sets. Examining the relationship between VGD and weekly, biweekly or monthly *T*_max_ and *T*_min_ at the locations of the 2,510 meteorological stations with >15 years of climatic data available for 1982–2011 (see Methods), we confirmed the statistically significant and negative partial correlations between VGD and *T*_max_ in the preceding 0–3 months at ∼43% of the stations, against only 14–16% of the stations between VGD and *T*_min_ for the same period (Supplementary Fig. 14). In addition, to determine whether the temporal binning of the GIMMS NDVI3g could bias the results, we performed the same analysis using VGD estimated from MODIS NDVI (2000–2010; Supplementary Fig. 15). For comparison, results from GIMMS NDVI3g during the same period (2000–2010) were also presented in Supplementary Fig. 15. The significantly negative correlation between VGD and preseason *T*_max_ was still unambiguously more prevailing (22% of area for GIMMS and 24% for MODIS) than that between VGD and *T*_min_ for the same period (8% of area for GIMMS and 12% for MODIS) although the results were not as apparent as that in Fig. 2 due to the shorter period in the MODIS NDVI time series (Supplementary Fig. 15).

### Temperature sensitivity of spring phenology

**Figure 3:**
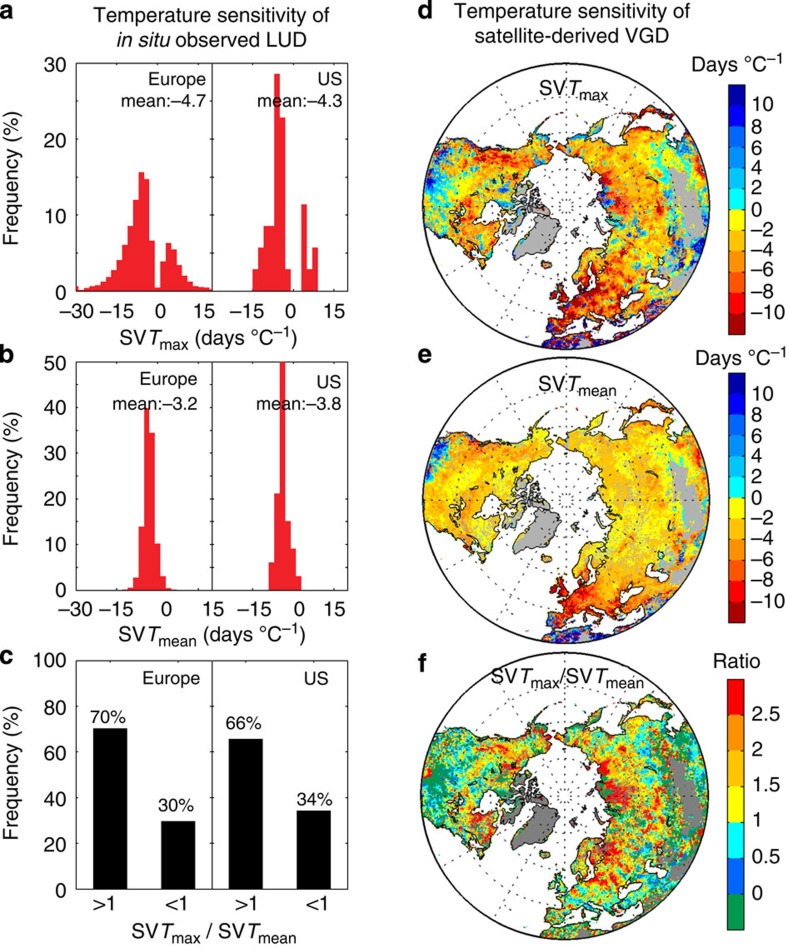
The sensitivity of LUD and VGD to *T*_max_ and *T*_mean_ during 1982–2011. The frequency distributions of the temperature sensitivity of LUD to (**a**) *T*_max_ (SV*T*_max_) and (**b**) *T*_mean_ (SV*T*_mean_) and (**c**) the ratio between SV*T*_max_ and SV*T*_mean_ (SV*T*_max_/SV*T*_mean_) in Europe and the United States. In the right panel of figure, the spatial distributions of the sensitivity of VGD are shown (**d**) *T*_max_ (SV*T*_max_) and (**e**) *T*_mean_ (SV*T*_mean_) and (**f**) the ratio between SV*T*_max_ and SV*T*_mean_ (SV*T*_max_/SV*T*_mean_) in the northern hemisphere during 1982–2011.

The stronger relationship between LUD (VGD) and *T*_max_ compared with *T*_min_ suggests that *T*_max_ is a better indicator of spring phenology. We therefore calculated the sensitivity (linear regression slope) of both *in situ*-observed LUD and satellite-derived VGD to preseason *T*_max_ (SV*T*_max_) using multiple linear regressions in which LUD (VGD) is regressed against *T*_max_, *T*_min_, precipitation and cloudiness (see Methods). On average, an increase of 1 °C in *T*_max_ would advance LUD by 4.7 days in Europe and 4.3 days in the United States (Fig. 3d,e) during 1982–2011. As for VGD, an increase in *T*_max_ of 1 °C was associated with a 3-day earlier VGD in the northern hemisphere during 1982–2011 (95% confidence interval: −12.0 days °C^−1^∼9.1days °C^−1^). The highest *T*_max_ sensitivities of VGD were observed in Europe, northern Siberia and northwestern Canada, where they exceeded −10 days °C^−1^ in some regions (Fig. 3a). Estimates of the *T*_max_ sensitivity of VGD were robust across the different satellite-derived VGD algorithms used (Supplementary Fig. 16). We note that the satellite-derived *T*_max_ sensitivity is not fully consistent with that derived from *in situ* observations at the same geographical locations (satellite pixel containing the site) in Europe (−8 ±7 versus −5±9 days °C^−1^) and in the United States (−3±3 versus −4±6 days °C^−1^). This small discrepancy between satellite and *in situ*-observation sensitivities may be due to their different temporal and spatial footprint. Compared with discrete, *in situ* data, GIMMS NDVI3g satellite observations provide more homogeneous phenological records over 8 by 8 km areas (including different species and sometimes different land cover types)^4^.

## Discussion

Our results show that both *in situ*-observed and satellite-derived spring leaf onset is more closely associated with the inter-annual variation of *T*_max_ than with *T*_min_. Three potential mechanisms may account for these results. First, plants in temperate and boreal regions need a critical level of forcing temperature (for example, GDD) to trigger spring phenology^13^. Only temperatures above this specific threshold (commonly set at 0 or 5 °C) count in GDD formation^11^,^18^. Before the onset of green-up, *T*_min_ is more likely to be below the threshold temperature than *T*_max_ and thus contribute less to fulfil the GDD requirement for green-up. This hypothesis is supported by the multi-year averaged values of *T*_max_ and *T*_min_ during *L*_max_ for VGD (Supplementary Fig. 17a,b). Averaged *T*_max_ was generally >5 °C, while averaged *T*_min_ remained below 0 °C in many areas of the northern hemisphere. Hence, daytime rather than nighttime warming in spring fulfills more efficiently the GDD requirement that triggers leaf onset. Second, photoperiod may also co-regulate spring phenology^19^. For example, the synchronicity of the daily cycles of light and temperature in spring makes daytime temperature an important determinant of *Arabidopsis* phenology^20^. The combined effects of photoperiod and daytime temperature in early spring could, hence, contribute to the stronger relationship with *T*_max_. Third, since most plant photosynthesis occurs during the daytime but is suspended during the nighttime, daytime temperature rather than nighttime temperature would be more responsible for plant carbon fixation and energy capture and thus produces a stronger effect on the onset of green-up.

In temperate dry regions, by contrast, a weak negative or even a positive interannual correlation between *T*_max_ and VGD is observed, which may be related to spring phenology being delayed by *T*_max_ regulated water stress. It has been suggested that spring phenology of temperate grasslands is co-determined by soil water availability and temperature^21^. Daytime warming is observed to reduce soil water content by enhancing evaporation^14^, which may partly or totally offset its advancing effect on VGD. On the other hand, significant negative correlations between *T*_min_ and VGD are observed in temperate dry regions (Fig. 2d), which could be partly attributed to decreased frost risk at higher nighttime temperature. It is also noted that in those areas the preseason average *T*_min_ is at 0 °C or above (Supplementary Fig. 17c) and thus *T*_min_ could contribute to fulfil the heat requirement for spring green-up. In addition, changes in plant community structure and composition in response to rising *T*_min_^22^ may also help explain this positive response of satellite-derived VGD to *T*_min_ variations, which need to be further tested.

Daily mean temperature (*T*_mean_) is currently used as the driver of spring phenology in models. Considering the unequal contribution of *T*_max_ versus *T*_min_ to spring leaf onset, models based on *T*_mean_ that includes an ineffective or less effective component of *T*_min_ may give questionable performance in analysing the responses of spring phenology to temperature changes, considering the recent faster warming rate at night than at daytime. For example, in Europe and in the United States, we found that the correlation between LUD and *T*_mean_ was weaker than that between LUD and *T*_max_ at >55% of the species–site combinations, although both the correlations were significant for comparable percentages of species–site combinations (Supplementary Fig. 18). Similarly, VGD in northern hemisphere also shows a weaker correlation with *T*_mean_ than with *T*_max_ in 65% of the study area, suggesting that *T*_max_ outperforms *T*_mean_ as a predictor of spring leaf onset variation. Furthermore, the absolute value of the LUD sensitivity to *T*_mean_, (see,for example, refs 3, 23), was smaller than its sensitivity to *T*_max_ in both Europe (−3.2 versus −4.7 days °C^−1^) and the United States (−3.8 versus −4.3 days °C^−1;^ Fig. 3d,e). The higher LUD sensitivity to *T*_max_ (SV*T*_max_) than to *T*_mean_ (SV*T*_mean_) obtained from *in situ* observations is also corroborated by satellite observations in 60% of the study area, with spatial variation in the magnitude of the positive differences between SV*T*_max_ and SV*T*_mean_ (Fig. 3c). A larger SV*T*_max_ than SV*T*_mean_ is found for the area north of 50°N compared with south of 50°N (Fig. 3c). The largest positive differences between SV*T*_max_ and SV*T*_mean_ were observed in Eastern Europe and north-central Siberia, where SV*T*_max_ was one to two times larger than SV*T*_mean_. On the other hand, regions where SV*T*_max_ is similar or even lower than SV*T*_mean_ are temperate dry ecosystems, where *T*_min_ rather than *T*_max_ is controlling the interannual variation of VGD as shown in Fig. 2.

**Figure 4:**
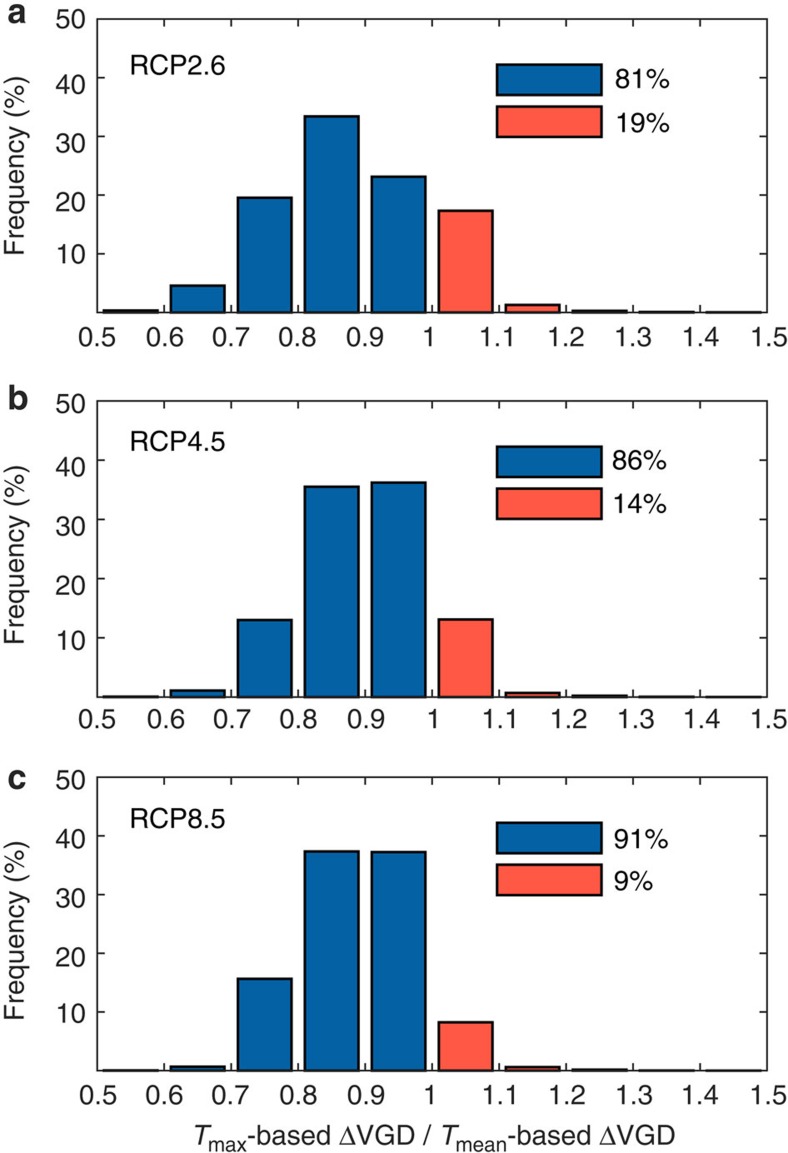
The ratios of future VGD changes predicted by a *T*_max_-based GDD concept model to that predicted by a *T*_mean_-based GDD concept model. Both *T*_mean_-based GDD approaches and *T*_max_-based GDD models were applied to predict the VGD changes (▵VGD) between 1991–2010 and 2081–2100, using 24 climate models and different climate change scenarios (RCP2.6, RCP4.5 and RCP8.5). For each RCP, the *T*_max_-based predictions and the *T*_mean_-based predictions were averaged across all models and the distributions of their ratio (*T*_max_-based predictions/*T*_mean_-based predictions) are shown in (**a**), (**b**) and (**c**). The ratio <1 (blue bar) represents that the future VGD changes predicted by *T*_mean_-based approaches are larger than those predicted by *T*_max_-based approaches and vice versa (red bar). The percentage of ratios <1 and the percentage of ratios >1 are both provided in **a**,**b** and **c**.

The findings suggest that spring phenology GDD models parameterized by daily mean temperature can be problematic in current vegetation models. To directly translate our findings into spring phenology predictions, we estimated and compared the changes of VGD under future climate and CO_2_ scenarios using *T*_mean_-based (as used in current vegetation models) and *T*_max_-based (proposed by this study) GDD models (see Methods). The scenarios include 24 climate models and three radiative forcing trajectories, RCP2.6, RCP4.5 and RCP8.5 (IPCC 2013). We found significant difference between *T*_max_- and *T*_mean_-based phenology predictions. The advance of VGD caused by warming was larger in the *T*_mean_-based prediction than in the *T*_max_-based one for 85% of the northern hemisphere across all the climate scenarios (Fig. 4 and Supplementary Fig. 19). This is because in all the climate models analysed, the projected increase of *T*_min_, and hence of *T*_mean_, is faster than that of *T*_max_ (IPCC 2013). This result suggests that *T*_mean_-based GDD models may overestimate changes in leaf onset, highlighting the need to incorporate the asymmetric phenology effects of daytime and nighttime temperature changes in earth system models.

In summary, our results provide information that can be used to improve the performance of current phenological module in DGVMs. The statistical analyses presented in this study, however, require more information for the accumulation of triggering energy and acclimation mechanisms. In this study, most of the preseason nighttime temperature does not contribute to the accumulation of a critical heat amount required for triggering spring phenology in the mid and high latitudes of the northern hemisphere. The heat requirement for spring green-up calculated based on daily mean temperature becomes problematic when one wants to predict future phenology changes based on past heat requirement, given the asymmetric warming rates between daytime and nighttime. While here we used maximum daytime and minimum nighttime temperature, which are not the same as the average daytime and nighttime temperature, our work suggests that temperature accumulation for spring green-up calculated at finer temporal resolutions, such as hourly or every 3 h, may be more appropriate. In addition, the impact of temperature on spring phenology has been found to be non-linear^24^, which further adds to the difficulty in using a statistical relationship established between current temperature and phenology to predict phenology under future climate scenarios. The non-linear impacts have been noticed and incorporated in early model development, such as the Spring Indices phenological models^25^. Finally, it should be noted that while daily weather can be a very random event, some synoptic-scale unusually warm daily events may also be critical in determining the timing of spring phenology^25^,^26^. The underlying mechanism through daytime and nighttime temperature affects spring phenology remains poorly understood in temperate and boreal ecosystems. Well-designed manipulation experiments therefore are needed to improve our understanding of the interaction between spring leaf unfolding phenology and daytime temperature, and ultimately result in more accurate simulations of spring phenology and better understanding of global carbon balance and ecosystem feedbacks to the ongoing climate change.

## Methods

### *In situ*-observation data set

We used *in situ* observations of LUD from two independent phenology data sets. One is the Pan European Phenological Database (PEP725; http://www.pep725.eu/ ), which is an open-access database with long-term plant phenological observations from 19,608 sites and 78 species across 25 European countries. This data set has been widely used for studying the relationships between spring phenology and climatic changes, especially global warming^8^,^27^. To exclude potential biases caused by outliers and inadequate degrees of freedom, we removed species–site compositions with the dates of LUD later than June (180 DOY: day of the year) and focused on the sites with >15 years records over the period 1982–2011. In total, 2,400 phenological sites and 24 plant species from PEP725 were used in this study. We also used *in situ* phenology observations from the USA National Phenology Network (USA–NPN; https://www.usanpn.org/results/data)^28^, and only shrubs of the *Lilac* genus had sufficient station records for LUD. Similarly, after excluding the data with the dates of LUD later than June (180 DOY) or with >15 years of records for 1982–2011, we analysed *Lilac* LUD data from 35 phenological sites in the United States. The distribution of selected phenological stations is shown in Supplementary Fig. S1. It should be noted that the first leafing date from USA–NPN was regarded as an equivalent of LUD here since USA–NPN does not include the exact phenological event of LUD as those defined in PEP725.

### Satellite-derived date of onset of green-up

The temporal cycle of NDVI is an indicator of the seasonal growth of vegetation and can be used for investigating vegetation phenology over large regions^1^,^29^,^30^. The GIMMS NDVI3g data set (1982–2011) with a spatial resolution of 1/12° and a 15-day interval has been used to monitor the phenological cycle of ecosystems^2^,^30^. Areas with sparse vegetation, that is, multi-year NDVIs <0.1, were excluded from the analyses. Using NDVI3g data sets, we applied four methods (Spline Midpoint, HANTS maximum, Polyfit maximum and Timesat SG) to estimate the VGD. Detailed information about the four VGD-deriving algorithms and the uncertainty in VGD estimation from the 15-day interval NDVI data set have been documented by refs 15, 30. Follow the previous study^15^, we applied a Bayesian constraint in each method to rule out the influence of snow cover and limit the VGD within the thermal growing season (5day average temperature >0 °C). The average VGDs of the four algorithms were used in this study (Supplementary Fig. 20), unless otherwise noted.

### Climatic data

Monthly data for *T*_max_, *T*_min_, *T*_mean_, precipitation and cloudiness were obtained from CRU TS 3.20 and are available for a regular 0.5° latitude/longitude grid for 1982–2011 (ref. 31). Due to the lack of solar-radiation data in the CRU data set during the study period, we used cloudiness data. To independently validate the results based on the CRU data set, we also used 0.5 × 0.5° latitude/longitude gridded 3-h climatic data applying WATCH Forcing Data Methodology to ERA-Interim data (WFDEI, 1982–2011)^32^, a 3-h global meteorological forcing data set (1982–2008)^33^ and the station-level global-surface summary of day product (GSOD) by the National Climatic Data Center. Daily weather can be a very random event, and the satellite data are of biweekly resolution, so the climatic data from WFDEI, Sheffield and GSOD were then rescaled to weekly, biweekly and monthly resolutions. For the GSOD data, we only considered the 2,510 stations with >15 years of available data for 1982–2011 and with NDVIs larger than 0.1 for the 0.5° latitude/longitude grids containing the stations. The data for short-wave radiation was obtained from WFDEI.

### Analyses

We used partial-correlation analyses to explore the effects of *T*_max_ and *T*_min_ on observed LUDs. With this approach, we could exclude the confounding effects of other climatic variables (precipitation and solar radiation) and of covariate effects between *T*_max_ and *T*_min_ (ref. 14). Temperature during the preseason dormancy period is arguably the most dominant factor for spring phenology^12^, and current phenology models in most DGVMs are solely based on temperature. It is therefore the aim of this study to identify the most appropriate temperature variables for use in phenology models. However, other environmental drivers in addition to temperature, such as precipitation, can also be involved in controlling the complex vegetation seasonality. Hence, in exploring the temperature effect on spring phenology, we have excluded the confounding effects of precipitation and cloudiness (radiation) in the partial-correlation analyses; while other variables such as soil moisture and soil temperature were not explicitly excluded, since temperature also indirectly influence them.

Spring phenological changes are highly associated with the temperatures in the preceding months^8^. To determine the length of the preseason whose average *T*_max_ had the largest influence on LUD, we calculated the partial-correlation coefficients between LUD and mean *T*_max_ during the 0, 1, 2, 3 … *k* months preceding LUD, controlling for corresponding average *T*_min_, accumulated precipitation and cloudiness (all variables non-detrended). The maximum *k* corresponded to the length of the period from the month of mean LUD (1982–2011) to the onset of preceding dormancy, defined as the month when the multi-year averaged mean temperature dropped to 0 °C, or November as a default value. The preceding months with the highest absolute partial-correlation coefficients were then considered as the *T*_max_-derived ‘preseason', in which *T*_max_ had the largest influence on the timing of green-up. Similarly, by replacing *T*_max_ with *T*_min_, we also obtained the *T*_min_-derived preseason.

To assess the robustness of our results, we also used grided climatic data sets at different temporal resolutions instead of the CRU monthly climatic data sets. In addition, to determine if winter chilling affected the responses of LUD to *T*_max_ and *T*_min_, we performed the same partial-correlation analysis with *T*_max_, *T*_min_, precipitation, cloudiness and winter temperature as independent variables. Winter temperature was defined as the average *T*_mean_ during the period from the onset of the preceding dormancy (the time at which daily mean temperature falls below 0 °C, or the default date of 1 November in the year preceding LUD) to the beginning of the *T*_max_ preseason.

Our results indicated that the interannual variation in LUD was more strongly associated with changes in *T*_max_ than that in *T*_min_, so we then only estimated the sensitivity of LUD to *T*_max_ based on multiple linear regressions with LUD as the dependent variable and *T*_max_, *T*_min_, precipitation and cloudiness as independent variables (all variables non-detrended). We used the monthly averaged values of each independent variable during the *T*_max_-derived preseason. We estimated the sensitivity of LUD to *T*_mean_ based on the same multiple linear regressions but replacing *T*_max_ and *T*_min_ with *T*_mean_. Accordingly, the monthly averaged values of each independent variable during the *T*_mean_-derived preseason were used in this analysis.

The same partial-correlation and sensitivity analyses were applied to satellite-derived observations, with preseason defined separately. To spatially match satellite data (1/12° spatial resolution) with climatic data (0.5° spatial resolution), we used averaged VGDs within each grid of the climatic data set. Besides the same robustness tests as those in the species–site level analysis (see above), we also performed additional robustness tests by using VGDs derived from individual algorithms instead of the multi-method averaged VGD, using station-level climate data set at different time resolution instead of CRU monthly climatic data sets, as well as using VGDs derived from MODIS NDVI instead of AVHRR NDVI estimated VGDs. The partial-correlation coefficients and temperature sensitivities derived from satellite and *in situ* observations were further compared with all the pixels covered by both the data sources.

### Future prospects

To directly translate our findings into spring phenology predictions, we performed phenology prediction tests with *T*_mean_ (used in current DGVMs) and *T*_max_ (proposed by this study) approaches, respectively. First, we calculated the mean GDD requirement for each pixel over the period of 1991–2010 using both daily *T*_max_ and *T*_mean_ from WFDEI climate data sets (Supplementary Fig. 21). The GDD requirement here is defined as an integration of temperature above 0 °C from 1 January to the satellite-derived VGD of each year. Second, we applied these two mean GDD values (GDD_*T*max_, GDD_*T*mean_) separately as the threshold to predict the VGD of each year over two periods, that is, 1991–2010 and 2081–2100, using 24 climate models and three climate scenarios (RCP2.6, RCP4.5 and RCP8.5). For each climate model and RCP scenario, the difference between the mean VGD of the two periods (mean_VGD_2081–2100_ minus mean_VGD_1991–2010_) was then calculated for both *T*_max_- and *T*_mean_-based GDD models. Finally, under each RCP scenario, the mean values of those differences across all climate models were calculated for each vegetated pixel. For comparison, we calculated the ratio of VGD changes predicted by GDD_*T*max_ to that predicted by GDD_*T*mean_. The spatial distribution of the ratios is shown in Supplementary Fig. 19.

## Author contributions

S. Piao designed research; J.T. and Q.L. performed analysis; S. Piao, J.T. and A.C. wrote the draft; and all the authors contributed to the interpretation of the results and the writing of the paper. S. Piao and J.T contributed equally to this work.

## Additional information

**How to cite this article:** Piao, S. *et al*. Leaf onset in the northern hemisphere triggered by daytime temperature. *Nat. Commun*. 6:6911 doi: 10.1038/ncomms7911 (2015).

## Supplementary Material

Supplementary Information Supplementary Figures 1-21

## References

[b1] Schwartz M. D., Ahas R. & Aasa A. Onset of spring starting earlier across the Northern Hemisphere. Glob. Change Biol. 12, 343–351 (2006).

[b2] Barichivich J. . Large-scale variations in the vegetation growing season and annual cycle of atmospheric CO_2_ at high northern latitudes from 1950 to 2011. Glob. Change Biol. 19, 3167–3183 (2013). 10.1111/gcb.12283 23749553

[b3] Richardson A. D. . Terrestrial biosphere models need better representation of vegetation phenology: results from the North American Carbon Program Site Synthesis. Glob. Change Biol. 18, 566–584 (2012).

[b4] Cleland E. E. . Shifting plant phenology in response to global change. Trends Ecol. Evol. 22, 357–365 (2007). 17478009 10.1016/j.tree.2007.04.003

[b5] CaraDonna P. J., Iler A. M. & Inouye D. W. Shifts in flowering phenology reshape a subalpine plant community. Proc. Natl Acad. Sci. USA 111, 4916–4921 (2014). 24639544 10.1073/pnas.1323073111 PMC3977233

[b6] Tylianakis J. M., Didham R. K., Bascompte J. & Wardle D. A. Global change and species interactions in terrestrial ecosystems. Ecol. Lett. 11, 1351–1363 (2008). 19062363 10.1111/j.1461-0248.2008.01250.x

[b7] Richardson A. D. . Climate change, phenology, and phenological control of vegetation feedbacks to the climate system. Agric. For. Meterol. 169, 156–173 (2013).

[b8] Menzel A. . European phenological response to climate change matches the warming pattern. Glob. Change Biol. 12, 1969–1976 (2006).

[b9] Keeling R. F., Piper S. C. & Heimann M. Global and hemispheric CO_2_ sinks deduced from changes in atmospheric O_2_ concentration. Nature 381, 218–221 (1996).

[b10] Kucharik C. J. . A multiyear evaluation of a Dynamic Global Vegetation Model at three AmeriFlux forest sites: vegetation structure, phenology, soil temperature, and CO_2_ and H_2_O vapor exchange. Ecol. Modell. 196, 1–31 (2006).

[b11] Harrington C. A., Gould P. J. & St. Clair J. B. Modeling the effects of winter environment on dormancy release of Douglas-fir. For. Ecol. Manage. 259, 798–808 (2010).

[b12] Hänninen H. & Kramer K. A framework for modelling the annual cycle of trees in boreal and temperate regions. Silva Fenn. 41, 167–205 (2007).

[b13] Chuine I. A unified model for budburst of trees. J. Theor. Biol. 207, 337–347 (2000). 11082304 10.1006/jtbi.2000.2178

[b14] Peng S. S. . Asymmetric effects of daytime and night-time warming on Northern Hemisphere vegetation. Nature 501, 88–92 (2013). 24005415 10.1038/nature12434

[b15] Fu Y. H. . Unexpected role of winter precipitation in determining heat requirement for spring vegetation green-up at northern middle and high latitudes. Glob. Change Biol. 20, 3743–3755 (2014). 10.1111/gcb.12610 24753114

[b16] IPCC. Contribution of Working Group I to the Fifth Assessment Report of the Intergovernmental Panel on Climate Change Cambridge Univ. Press (2013).

[b17] Jeong S. J., Ho C. H., Gimi H. J. & Brown M. E. Phenology shifts at start vs. end of growing season in temperate vegetation over the Northern Hemisphere for the period 1982–2008. Glob. Change Biol. 17, 2385–2399 (2011).

[b18] Hänninen H. Modelling bud dormancy release in trees from cool and temperate regions. Acta For. Fenn 213, 1–47 (1990).

[b19] Körner C. & Basler D. Phenology under global warming. Science 327, 1461–1462 (2010). 20299580 10.1126/science.1186473

[b20] Chew Y. H. . An augmented *Arabidopsis* phenology model reveals seasonal temperature control of flowering time. N. Phytol. 194, 654–665 (2012). 10.1111/j.1469-8137.2012.04069.x 22352314

[b21] Yu F., Price K. P., Ellis J. & Shi P. Response of seasonal vegetation development to climatic variations in eastern central Asia. Remote Sens. Environ. 87, 42–54 (2003).

[b22] Alward R. D., Detling J. K. & Milchunas D. G. Grassland vegetation changes and nocturnal global warming. Science 283, 229–231 (1999). 9880257 10.1126/science.283.5399.229

[b23] Wolkovic E. M. . Warming experiments underpredict plant phenological responses to climate change. Nature 485, 494–497 (2012). 22622576 10.1038/nature11014

[b24] Pope K. S. . Detecting nonlinear response of spring phenology to climate change by Bayesian analysis. Glob. Change Biol. 19, 1518–1525 (2013). 10.1111/gcb.12130 23505006

[b25] Schwartz M. D., Ahas R. & Aasa A. Onset of spring starting earlier across the northern hemisphere. Glob. Change Biol. 12, 343–351 (2006).

[b26] Schwartz M. D. & Marotz G. A. Synoptic events and spring phenology. Phys. Geogr. 9, 151–161 (1988).

[b27] Cook B. I. . Sensitivity of spring phenology to warming across temporal and spatial climate gradients in two independent databases. Ecosystems 15, 1283–1294 (2012).

[b28] Schwartz M. D., Betancourt J. L. & Weltzin J. F. From Caprio's lilacs to the USA National Phenology Network. Front. Ecol. Environ. 10, 324–327 (2012).

[b29] Piao S. L. . Evidence for a weaking relationship between interannual temperature variability and northern vegetataion activity. Nat. Commun. 5, 5018 (2014). 25318638 10.1038/ncomms6018

[b30] White M. A. . Intercomparison, interpretation, and assessment of spring phenology in North America estimated from remote sensing for 1982-2006. Glob. Change Biol. 15, 2335–2359 (2009).

[b31] Mitchell T. D. & Hulme M. New, climate data for political areas. Area 34, 109–112 (2002).

[b32] Weedon G. P. . The WFDEI meteorological forcing dataset: WATCH forcing data methodology applied to ERA-Interim reanalysis data. Water Resour. Res. 50, 7505–7514 (2014).

[b33] Sheffield J. G. & Wood E. F. Development of a 50-yr high-resolution global dataset of meteorological forcings for land surface modeling. J. Climate 19, 3088–3111 (2006).

