# Supplementary material for: Leaf onset in the northern hemisphere triggered by daytime temperature
Source: Nat Commun. 2015 Apr 23;6:6911. doi: 10.1038/ncomms7911 (PMC4423217; doi:10.1038/ncomms7911)
Supplement: Supplementary Information — Supplementary Figures 1-21 [file ncomms7911-s1.pdf]

Supplementary Figures

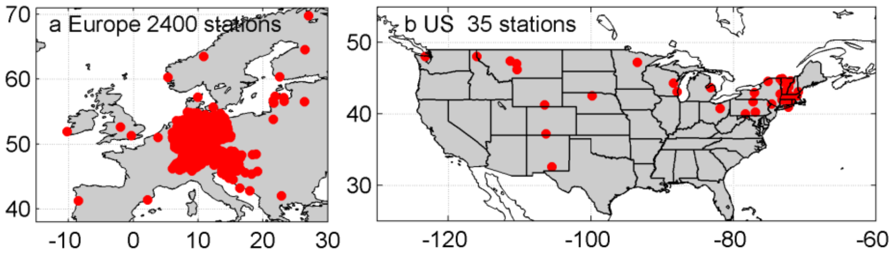

Supplementary Figure 1: The spatial distributions of in situ phenology sites in Europe (a) and US (b).

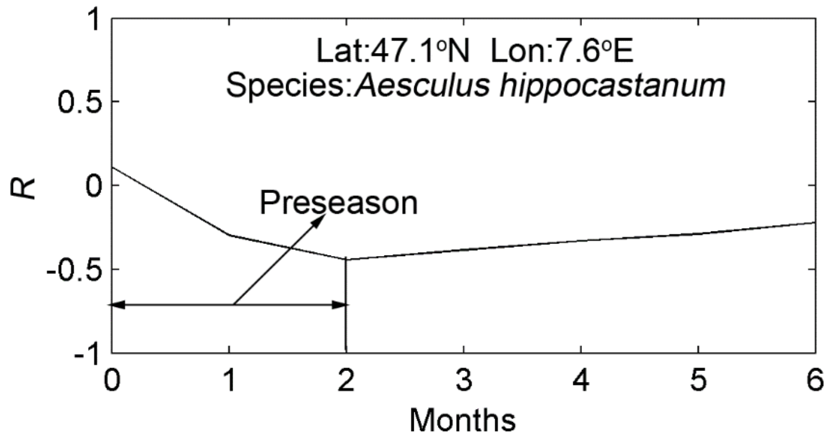

Supplementary Figure 2: **An example of the length of the "preseason" using *Aesculus hippocastanum* at the specific site where the coordinate is provided.**

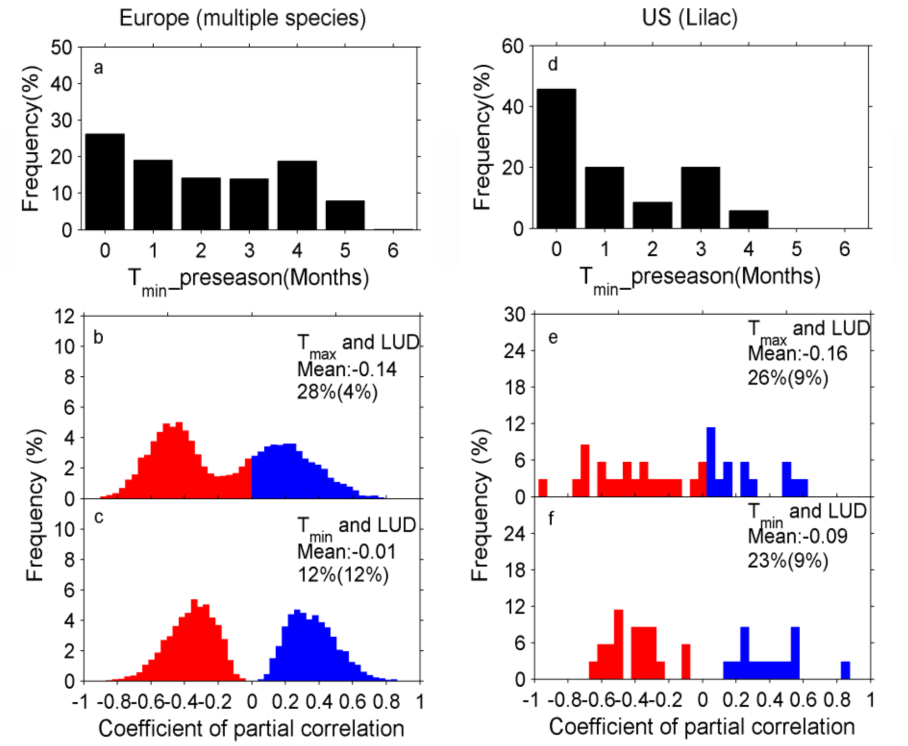

Supplementary Figure 3: Distribution of the partial correlation coefficients between leaf unfolding date (LUD) and T_{min}-preseason T_{max} (b and e) and T_{min} (c and f) in the Europe and US during 1982-2011. The frequency distributions of the length (in months) of T_{min}_preseason are shown in (a) Europe and (d) the US. The mean values of significant (P<0.05) partial-correlation coefficients across all phenological stations, the percentages of significantly negative partial correlations and the percentages of significantly positive partial correlations (in parentheses) are provided in (b), (c), (e) and (f).

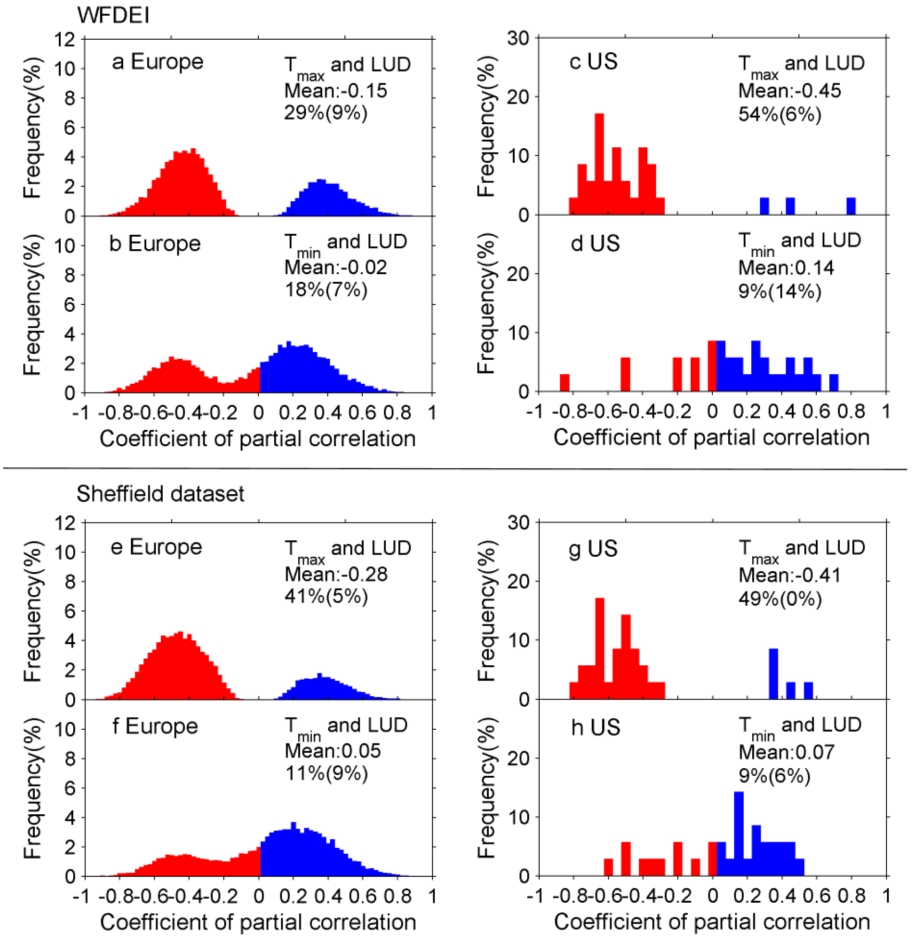

Supplementary Figure 4: Distribution of the partial correlation coefficients between LUD and weekly Tmax-preseason T_{max} and weekly T_{min} in Europe (left panels) and the US (right panels) during 1982-2011. Here the climate data are obtained from WFDEI (a-d) and Sheffield et al (e-h). The mean values of significant ($P<0.05$) partial-correlation coefficients across all phenological stations, the percentages of significantly negative partial correlations and the percentages of significantly positive partial correlations (in parentheses) are provided in each sub-panels.

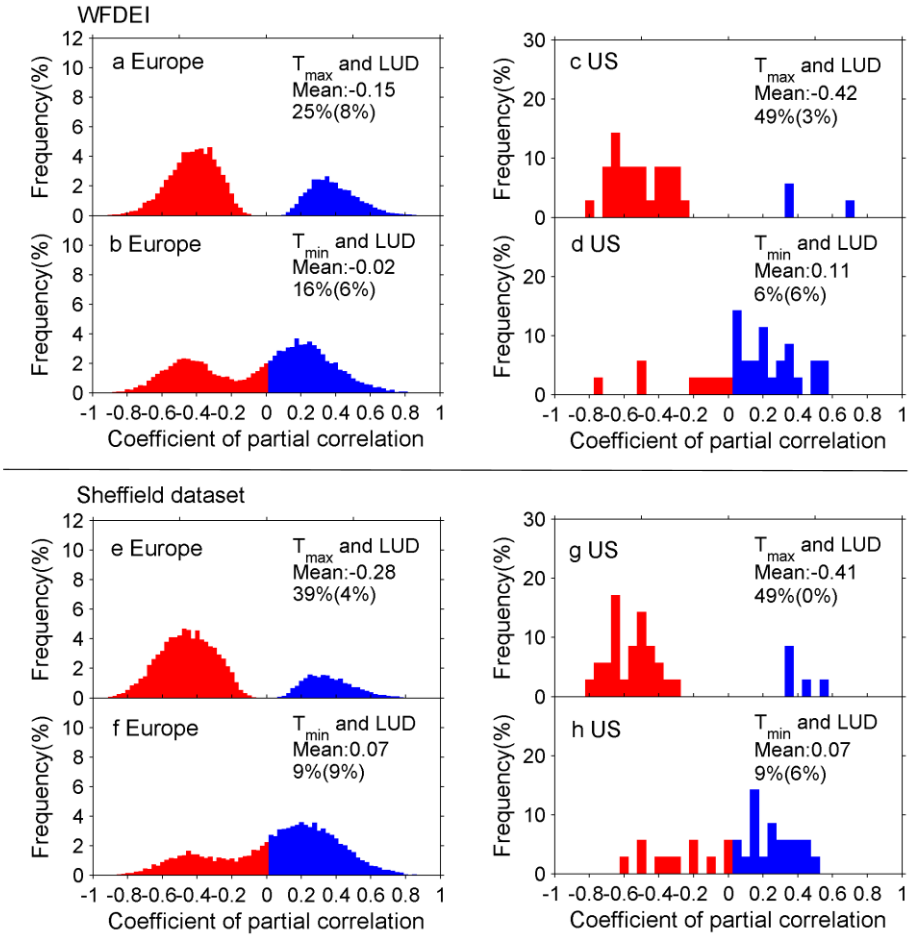

Supplementary Figure 5: Distribution of the partial correlation coefficients between LUD and biweekly T_{max}-preseason T_{max} and biweekly T_{min} in Europe (left panels) and the US (right panels) during 1982-2011. Here the climate data are obtained from WFDEI (a-d) and Sheffield et al (e-h). The mean values of significant ($P<0.05$) partial-correlation coefficients across all phenological stations, the percentages of significantly negative partial correlations and the percentages of significantly positive partial correlations (in parentheses) are provided in each sub-panels.

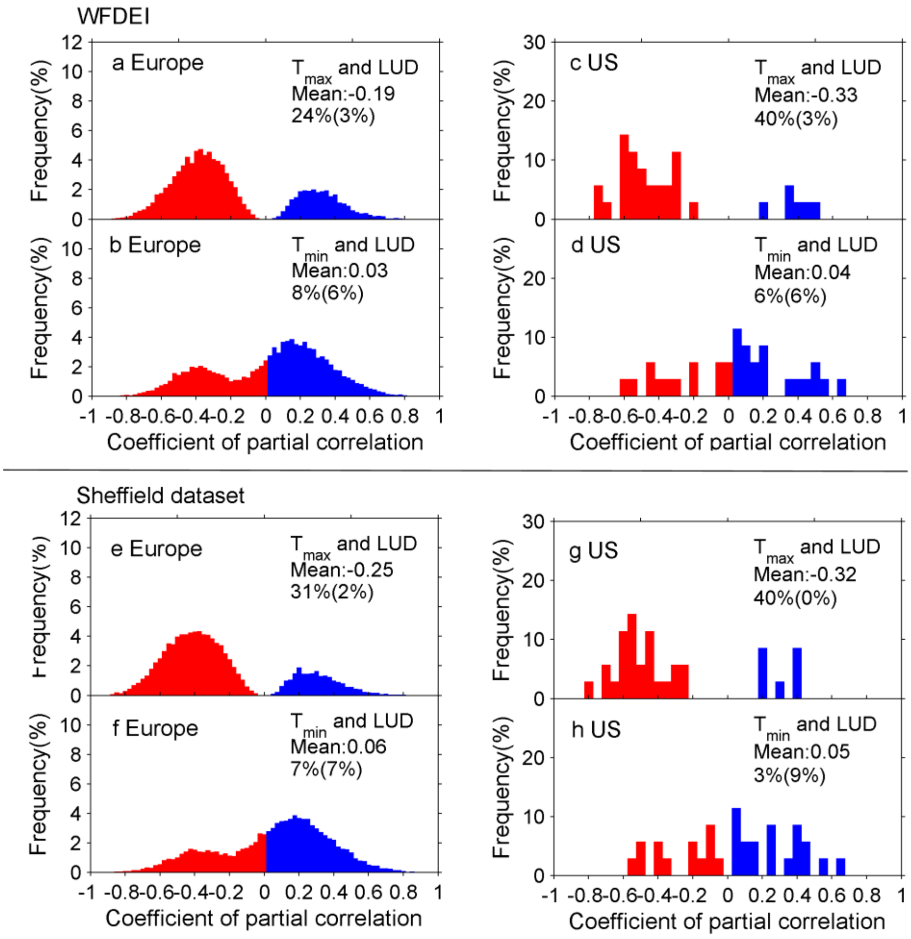

Supplementary Figure 6: Distribution of the partial correlation coefficients between LUD and monthly T_{max}-preseason T_{max} and monthly T_{min} in Europe (left panels) and the US (right panels) during 1982-2011. Here the climate data are obtained from WFDEI (a-d) and Sheffield et al (e-h). The mean values of significant ($P<0.05$) partial-correlation coefficients across all phenological stations, the percentages of significantly negative partial correlations and the percentages of significantly positive partial correlations (in parentheses) are provided in each sub-panels.

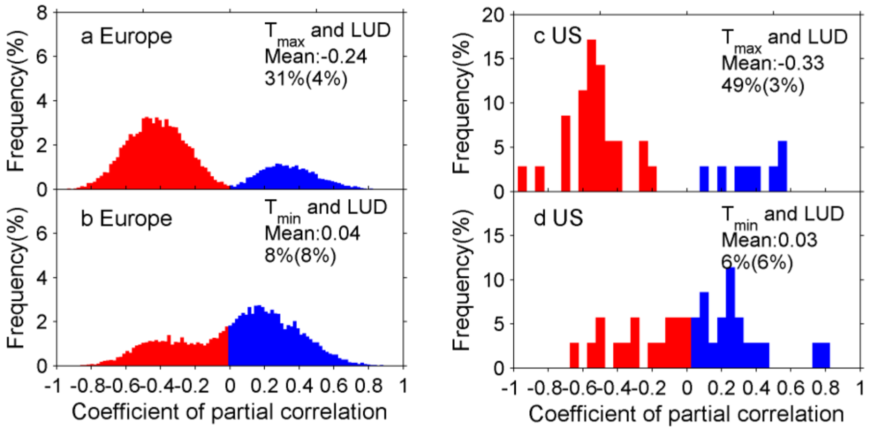

Supplementary Figure 7: Distribution of partial correlation coefficientbetween T_{max}**-preseason mean** T_{max} **(a, c),** T_{min} **(b, d) and LUD after controlling for corresponding** T_{min} **(or** T_{max}**), cloudiness, precipitation, and preceding winter temperature.** Winter temperature was defined as the average T_{mean} during the period from the onset of the preceding dormancy (the time at which daily mean temperature falls below 0 ℃, or the default date of 1 November in the year preceding LUD) to the beginning of the T_{max}-preseason.

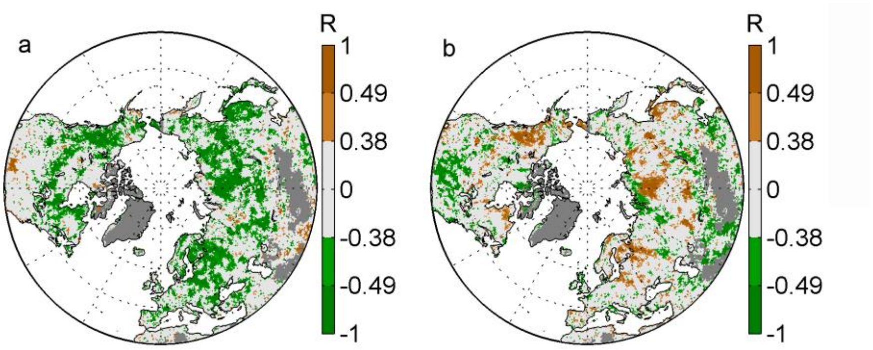

Supplementary Figure 8: Spatial distribution of the partial correlation coefficients between onset dates of vegetation green–up (VGD) and T_{min}-preseason T_{max} and T_{min} in the Northern Hemisphere during 1982-2011. a, Partial correlation coefficient (R) between T_{max} and VGD during T_{min}-derived preseason after controlling for corresponding T_{min}, cloudiness and precipitation. b, Partial correlation coefficient (R) between T_{min} and VGD during T_{min}-derived preseason after controlling for corresponding Tmax, cloudiness and precipitation.

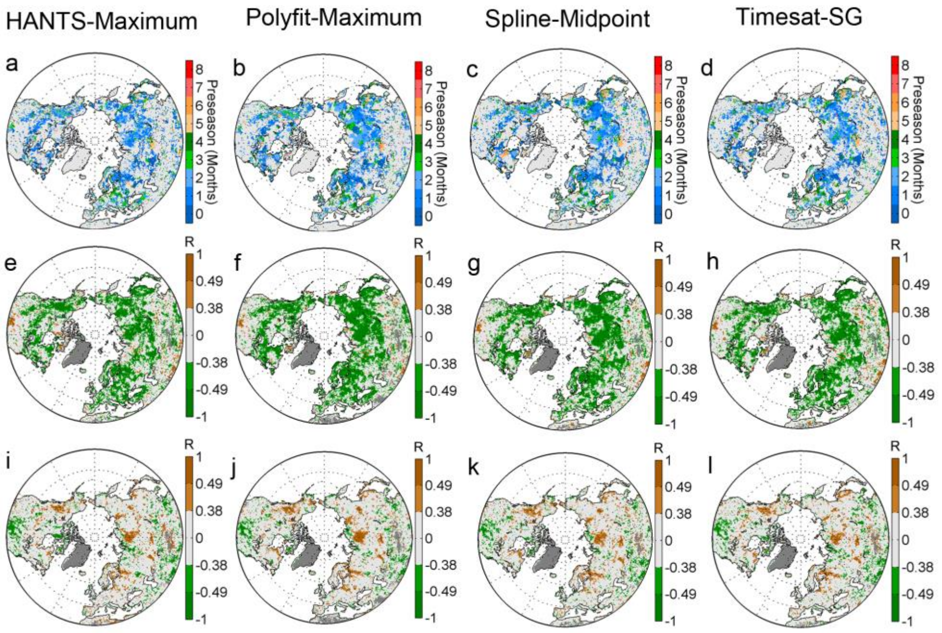

Supplementary Figure 9: Spatial distribution of the partial correlation coefficients between VGD and T_{max}-preseason T_{max} or T_{min} in the Northern Hemisphere during 1982-2011. Different from Figure 2 where multi-method averaged VGD is used, here we use VGD derived from each different method. **a**, **b**, **c**, and **d** show the length (in month) of preseason for each method. **e**, **f**, **g**, and **h** show the correlation coefficient (R) between preseason T_{max} and VGD for each method. Partial correlation coefficient (R) between T_{min} during the T_{max}-preseason and VGD derived from each method are shown in **i**, **j**, **k**, and **l**.

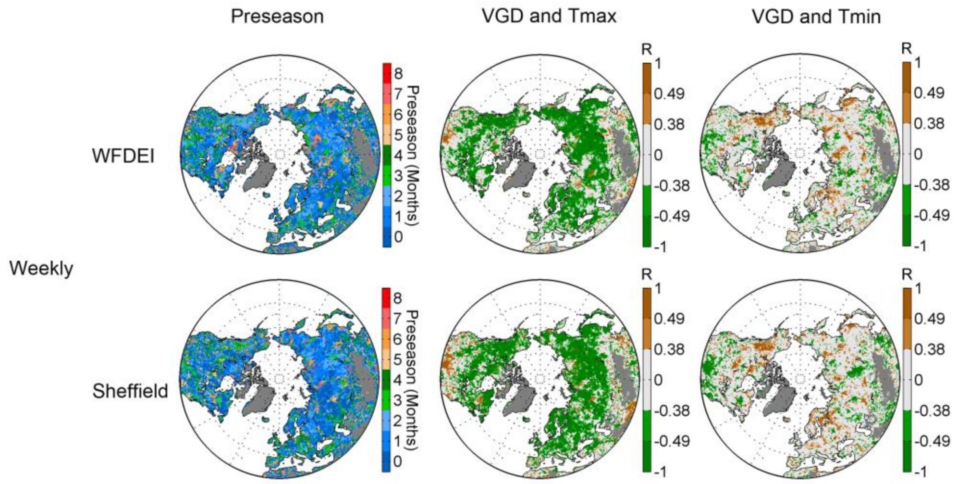

Supplementary Figure 10: Spatial distribution of the partial correlation coefficients between VGD and weekly T_{max}-preseason T_{max} and weekly T_{max}-preseason T_{min} in Northern Hemisphere during 1982-2011. Here the climate data are obtained from WFDEI (upper panels) and Sheffield et al (lower panels). The left-most panels demonstrate the length of T_{max}-preseason, the middle panels demonstrate the partial correlation coefficients between VGD and T_{max}-preseason T_{max} and the right-most panels demonstrate the partial correlation coefficients between VGD and T_{max}-preseason T_{min}.

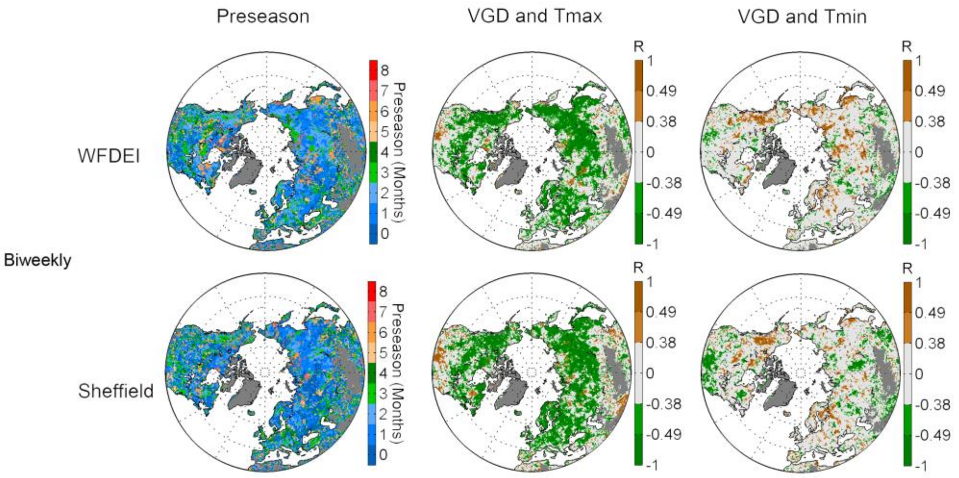

Supplementary Figure 11: Spatial distribution of the partial correlation coefficients between VGD and biweekly T_{max}-preseason T_{max} and biweekly T_{max}-preseason T_{min} in Northern Hemisphere during 1982-2011. Here the climate data are obtained from WFDEI (upper panels) and Sheffield et al (lower panels). The left-most panels demonstrate the length of T_{max}-preseason, the middle panels demonstrate the partial correlation coefficients between VGD and T_{max}-preseason T_{max} and the right-most panels demonstrate the partial correlation coefficients between VGD and T_{max}-preseason T_{min}.

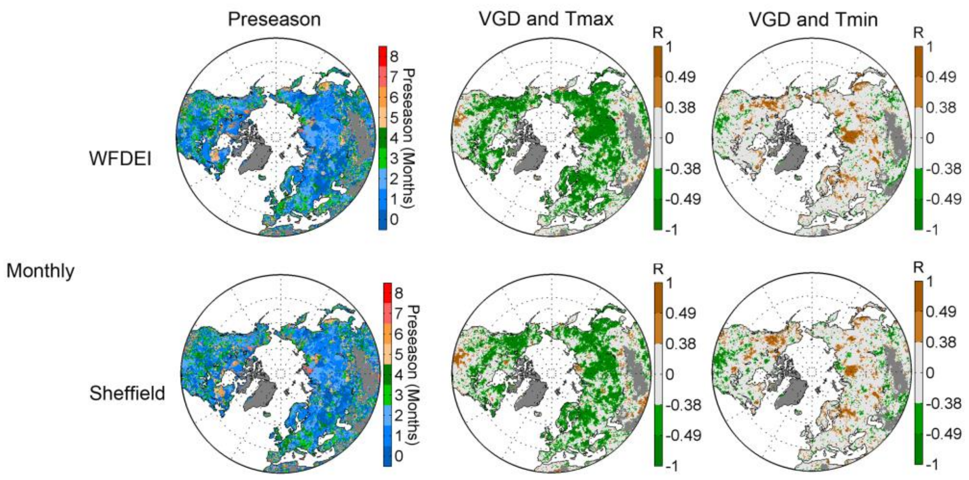

Supplementary Figure 12: Spatial distribution of the partial correlation coefficients between VGD and monthly T_{max}-preseason T_{max} and monthly T_{max}-preseason T_{min} in Northern Hemisphere during 1982-2011. Here the climate data are obtained from WFDEI (upper panels) and Sheffield et al (lower panels). The left-most panels demonstrate the length of T_{max}-preseason, the middle panels demonstrate the partial correlation coefficients between VGD and T_{max}-preseason T_{max} and the right-most panels demonstrate the partial correlation coefficients between VGD and T_{max}-preseason T_{min}.

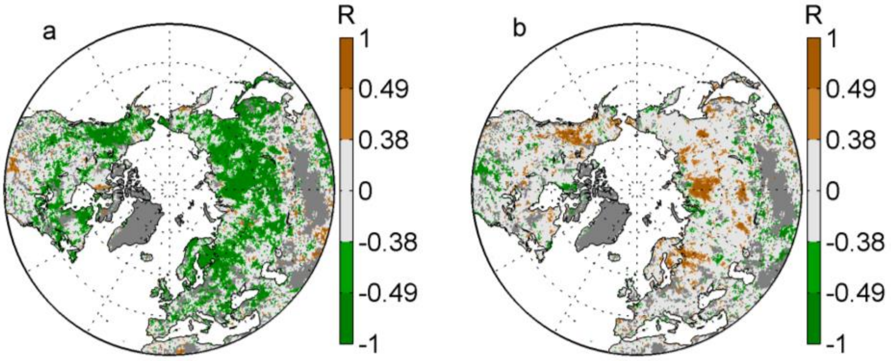

Supplementary Figure 13: Spatial distribution of partial correlation coefficient between T_{max}-preseason mean T_{max} (a), T_{min} (b)and VGD after controlling for corresponding T_{min} (or T_{max}), cloudiness, precipitation, and preceding winter temperature. Winter temperature was defined as the average T_{mean} during the period from the onset of the preceding dormancy (the time at which daily mean temperature falls below 0 ℃, or the default date of 1 November in the year preceding LUD) to the beginning of the T_{max}-preseason.

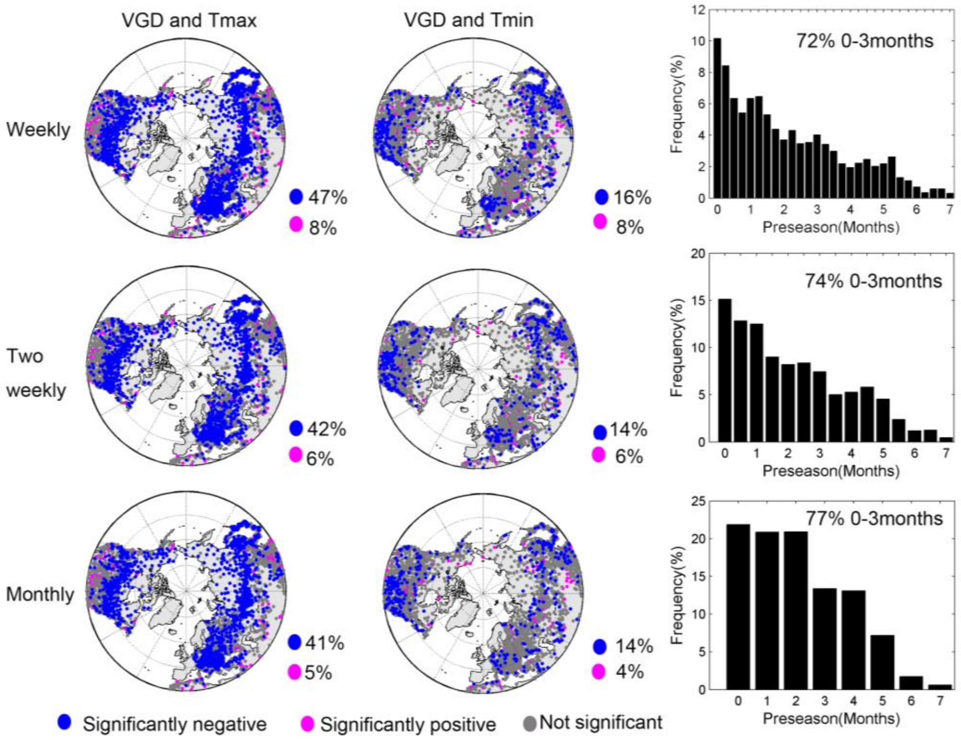

Supplementary Figure 14: Spatial distribution of the partial correlation coefficients between VGD and T_{max}-preseason T_{max} (left panels) and T_{min} (right panels) across climate stations for three time resolutions, i.e. weekly, two weekly and monthly, in Northern Hemisphere during 1982-2011. Here the climate data are obtained from GSOD. The histograms show the distribution of the length (in months) of T_{max}-preseason.

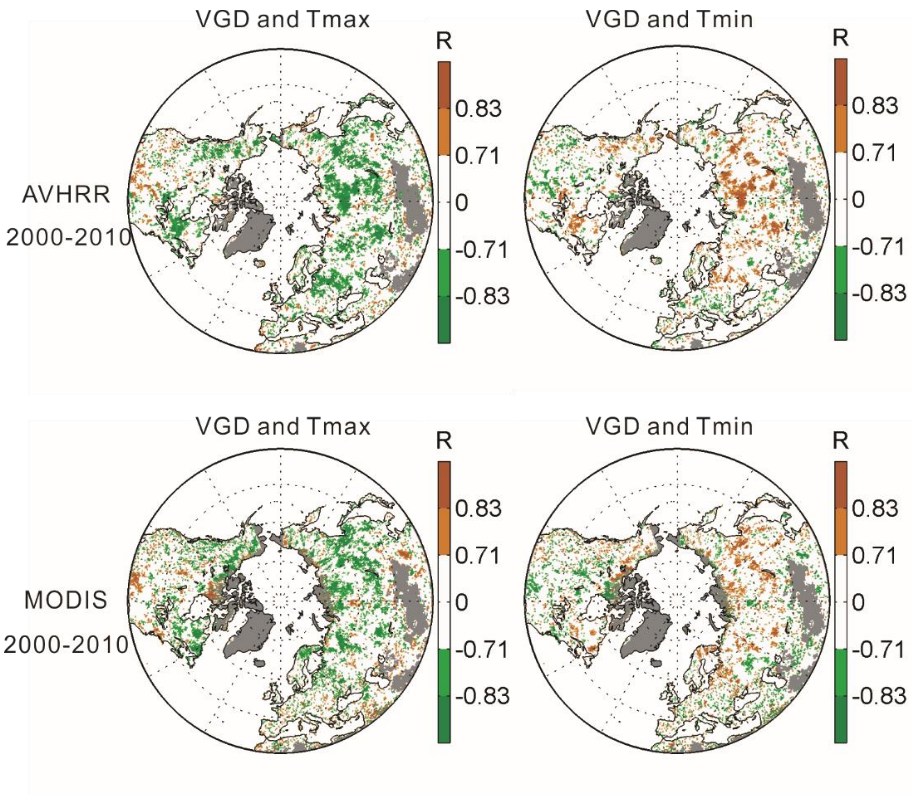

Supplementary Figure 15: Spatial distribution of the partial correlation coefficient between onset dates of vegetation green-up (VGD) and monthly T_{max}-preseason T_{max} (left panels) and T_{min} (right panels) in the Northern Hemisphere during 2000-2010 using both AVHRR (upper panels) and MODIS data set (lower panels).

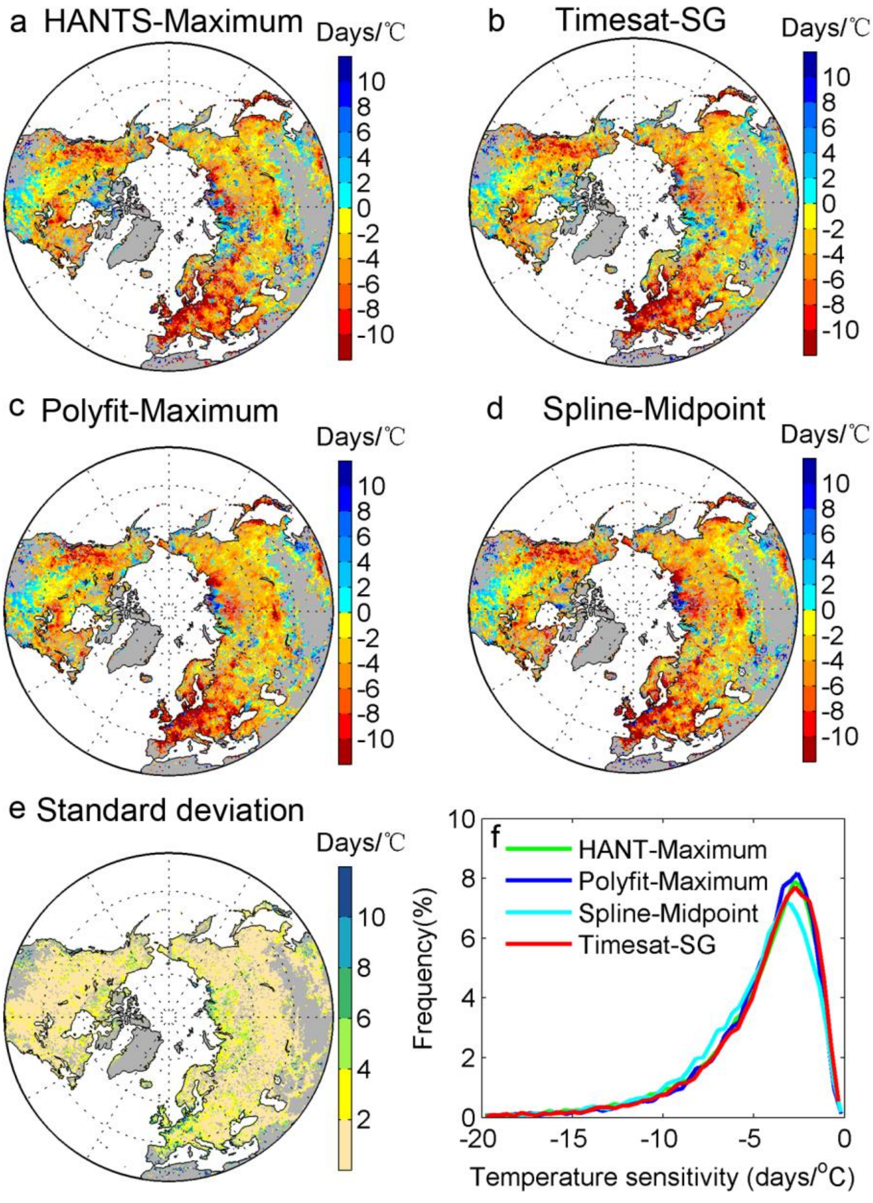

Supplementary Figure 16: Spatial distribution of the sensitivity of VGD to T_{max}**-preseason T_{max} in the Northern Hemisphere during 1982-2011.** Here **a**, **b**, **c**, and **d** show the results from four different algorithms in deriving VGDs. Only areas with a significantly (P<0.05) correlation with T_{max} are shown. **f** shows the frequency distribution of sensitivities derived from each algorithm.

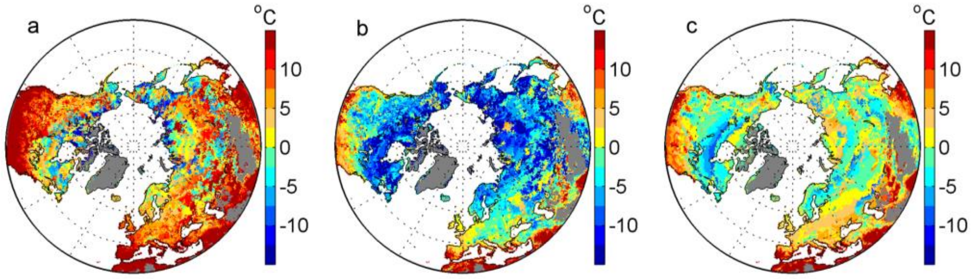

Supplementary Figure 17: Spatial patterns of multi-year averaged T_{max}-preseason T_{max} and T_{min} (1982-2011). a and **b** show the spatial pattern of multi-year averaged T_{max} and T_{min} during the T_{max}-preseason that was defined as the period before VGD for which the partial-correlation coefficient between VGD and average T_{max} is the largest. **c**, the spatial pattern of multi-year averaged T_{min} during the month when multi-year averaged VGD occurs.

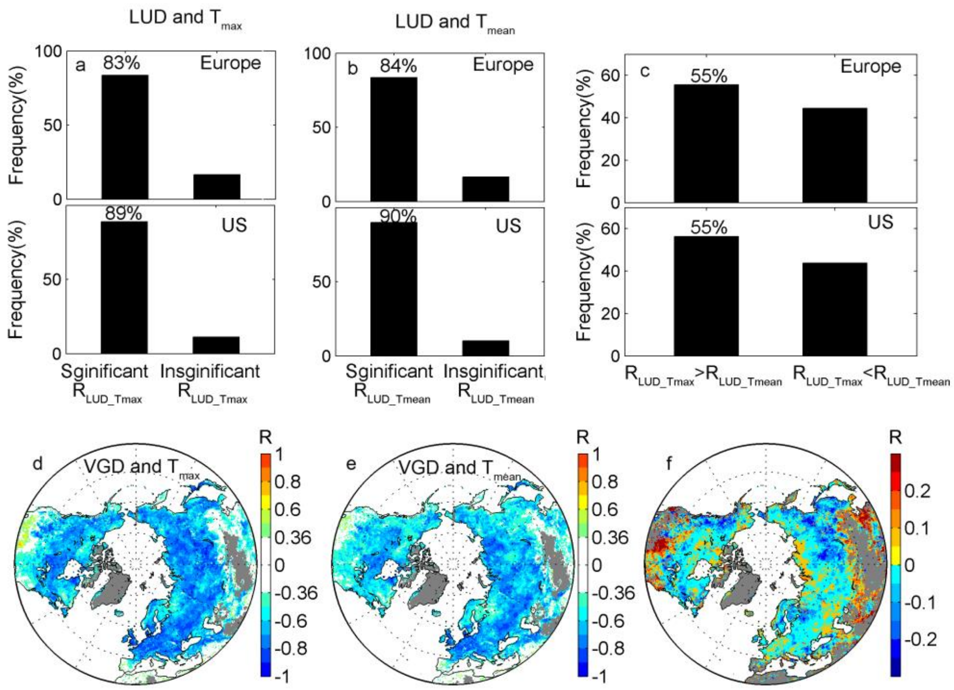

Supplementary Figure 18: Spatial patterns of the partial correlation coefficients between LUD (VGD) and T_{max} and T_{mean} (1982-2011). a, Percentage of significant and insignificant partial correlation coefficients between LUD and T_{max} in Europe and the US. **b**, Percentage of significant and insignificant partial correlation coefficients between LUD and T_{mean} in Europe and the US. **c**, The percentage of species-site combinations where the partial correlation coefficients shown in **a** is larger and smaller than the partial correlation coefficients shows in **b** for both Europe and US. **d**, Spatial patterns of the correlation coefficients between VGD and T_{max}. **e**, Spatial patterns of the correlation coefficients between VGD and T_{mean}. **f**, The differences of the correlation coefficients shown in **d** and **e** . It should be noted that only corresponding precipitation and cloudiness have been controlled for in all the mentioned partial correlation analysis.

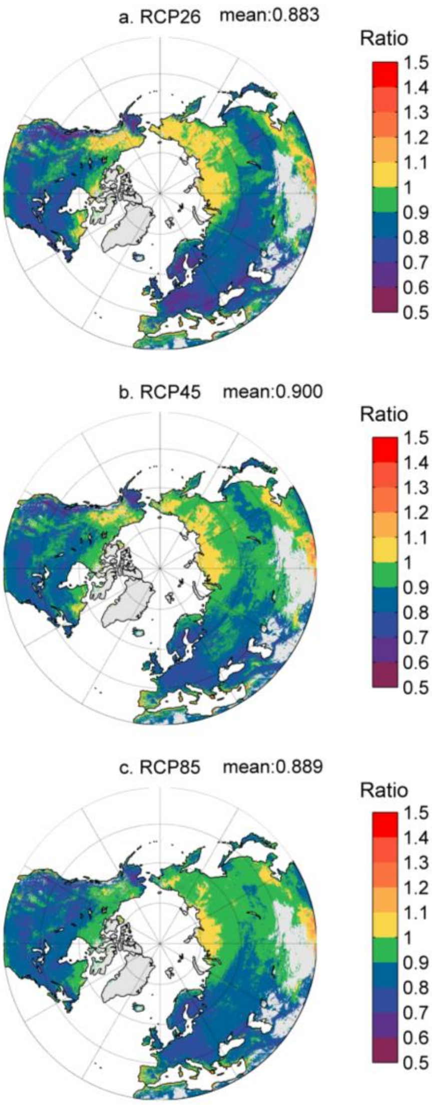

Supplementary Figure 19: The spatial pattern of the ratio of mean vegetation green-up date (VGD) changes predicted by GDD_{Tmax} to mean VGD changes predicted by GDD_{Tmean} under three climate change scenarios, i.e. RCP2.5 (a), RCP4.5 (b) and RCP 8.5 (c). The mean VGD changes were calculated between two mean VGDs over the two periods 2081-2100 and 1991-2010 (Mean_VGD2081-2100 minus Mean_VGD1991-2010). The mean values of the ratios were provided.

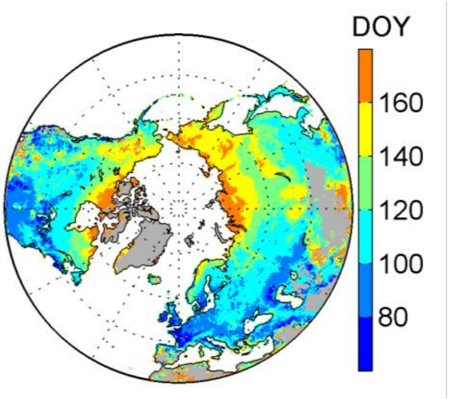

Supplementary Figure 20: Spatial patterns of the multi-year averaged onset dates of vegetation green up (VGD, 1982-2011). VGD used here is the average of four different VGD algorithms, (see Methods). DOY means day of the year.

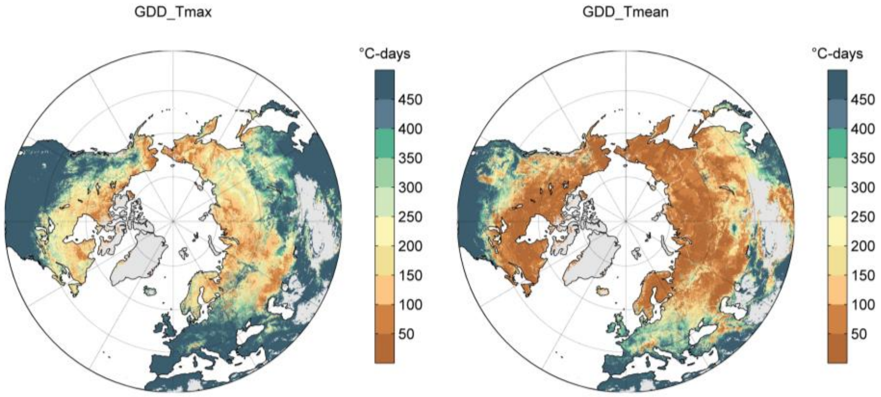

Supplementary Figure 21: The spatial pattern of mean growing degree-days (GDD) requirement for vegetation green-up date (VGD) over the period 1991-2010 using daily T_{max} (left panel) and T_{mean} (right panel) from WFDEI climate datasets. GDD requirement is defined as an integration of T_{max} and T_{mean}, respectively, above 0°C from 1 January to the satellite derived VGD of each year.
